# BUHO: A MATLAB Script for the Study of Stress Granules and Processing Bodies by High-Throughput Image Analysis

**DOI:** 10.1371/journal.pone.0051495

**Published:** 2012-12-20

**Authors:** Marcelo Perez-Pepe, Victoria Slomiansky, Mariela Loschi, Luciana Luchelli, Maximiliano Neme, María Gabriela Thomas, Graciela Lidia Boccaccio

**Affiliations:** 1 Fundación Instituto Leloir, Buenos Aires, Argentina; 2 Instituto de Investigaciones Bioquímicas Buenos Aires-Consejo Nacional de Investigaciones Científicas y Tecnológicas, Buenos Aires, Argentina; 3 Facultad de Ciencias Exactas y Naturales, University of Buenos Aires, Buenos Aires, Argentina; German Cancer Research Center, Germany

## Abstract

The spontaneous and reversible formation of *foci* and filaments that contain proteins involved in different metabolic processes is common in both the nucleus and the cytoplasm. Stress granules (SGs) and processing bodies (PBs) belong to a novel family of cellular structures collectively known as mRNA silencing *foci* that harbour repressed mRNAs and their associated proteins. SGs and PBs are highly dynamic and they form upon stress and dissolve thus releasing the repressed mRNAs according to changes in cell physiology. In addition, aggregates containing abnormal proteins are frequent in neurodegenerative disorders. In spite of the growing relevance of these supramolecular aggregates to diverse cellular functions a reliable automated tool for their systematic analysis is lacking. Here we report a MATLAB Script termed BUHO for the high-throughput image analysis of cellular *foci*. We used BUHO to assess the number, size and distribution of distinct objects with minimal deviation from manually obtained parameters. BUHO successfully addressed the induction of both SGs and PBs in mammalian and insect cells exposed to different stress stimuli. We also used BUHO to assess the dynamics of specific mRNA-silencing *foci* termed Smaug 1 *foci* (S-*foci*) in primary neurons upon synaptic stimulation. Finally, we used BUHO to analyze the role of candidate genes on SG formation in an RNAi-based experiment. We found that FAK56D, GCN2 and PP1 govern SG formation. The role of PP1 is conserved in mammalian cells as judged by the effect of the PP1 inhibitor salubrinal, and involves dephosphorylation of the translation factor eIF2α. All these experiments were analyzed manually and by BUHO and the results differed in less than 5% of the average value. The automated analysis by this user-friendly method will allow high-throughput image processing in short times by providing a robust, flexible and reliable alternative to the laborious and sometimes unfeasible visual scrutiny.

## Introduction

An emerging concept in cell biology is the formation of microscopically visible supramolecular assemblies involved in very distinct cellular processes, ranging from metabolism to RNA silencing. Noxious insults that activate the cellular stress response trigger the transient accumulation of stress granules (SGs) in the cytoplasm. SGs belong to a novel family of cellular structures collectively known as mRNA silencing *foci* that harbour repressed mRNAs and their associated proteins [1]. SGs assemble as a consequence of the global translation silencing provoked by the inactivation of the translation initiation factor 2α (eIF2α) by specific kinases that are activated upon cell stress. Processing Bodies (PBs) are related *foci* and are as well induced upon stress. There is controversy on whether SGs and PBs are cause or consequence of mRNA repression. SGs and PBs from different organisms appear to differ on composition and function and a continuous spectrum of entities exits. Nevertheless, SGs and PBs are highly dynamic and they may dissolve thus releasing the repressed mRNAs to allow translation according to cellular needs [2], [3], [4], [5], [6]. SGs contribute to the cell survival response by regulation of specific signalling pathways [7], and the cellular mechanisms that control their assembly and disassembly are incipiently described (reviewed in [1]).

Cell damage induces the assembly of additional supramolecular complexes in both the nucleus and in the cytoplasm. The endoribonuclease Inositol-requiring enzyme 1 (Ire-1) and UV-damaged RNA concentrate in cytoplasmic Ire-1 *foci* or UV-bodies, respectively. DNA replication factories form *foci* associated to damaged DNA [8], [9], [10], [11], [12], [13]. Translational silencing is frequently linked to the formation of distinct aggregates. For instance, local translation regulation is relevant to synaptic activity and involves the dynamic formation of a plethora of mRNA silencing *foci* located at the synapse [14], [15]. The eukaryotic initiation factor 2B (eIF2B) bodies are another example linked to translation regulation and their integrity and dynamics are crucial for eIF2 recycling [16], [17], [18]. Finally, a number of dynamic supramolecular factories were reported to occur in several cell types. These include Purinosomes and glutamine synthetase *foci*, which concentrate specific biosynthetic enzymes and dissolve when the levels of the end-product metabolite increase [19], [20], [21]. Enzymes may also reversibly aggregate for storage in an inactive state, as is the case of the CTP synthetase in yeast cells, *Drosophila* embryo and mammalian axons [22]. In addition to all these structures, a large variety of cytoplasmic or nuclear supramolecular complexes such as nuclear stress bodies, eisosomes [23], U-bodies, splicing speckles and Cajal bodies, among others, assemble and disassemble dynamically, depending on changes in cell physiology (reviewed in [1], [24]).

Besides its relevance in mRNA silencing and decay, SGs and PBs are relevant to the pathophysiology of several conditions. SG and PB dynamics are affected by virus infections (reviewed in [25], [26], [27]). More recently, SGs have been linked to abnormal protein aggregates involved in neurodegenerative diseases [28], [29], [30], [31]. Thus, there is an increasing interest in identifying pharmacological and genetic enhancers and inhibitors of SG and PB formation. A pioneer work for identifying regulators of SGs and PBs involved the analysis of thousands of micrographs [32]. These high-throughput studies would be facilitated by automated methods. Here we report a MATLAB script that we named BUHO for the analysis of supramolecular aggregates in mammalian and *Drosophila* cells. Briefly, objects were identified by their similarity to a number of prototypes, which were of different size, shape and intensity. BUHO is easy to use and simple to adjust for the analysis of a variety of cellular components. We used it to analyze SG and PB induction in mammalian and *Drosophila* cells exposed to stress. With minor adjustments, the script was also useful to study the dynamics of specific neuronal mRNA silencing *foci* termed S-*foci*. Finally, we used BUHO to analyze the effect of candidate genes on SG formation in an RNAi-based experiment in *Drosophila* S2R+ cells. We found that focal adhesion kinase 56D (FAK56D), general control non-derepresible 2 (GCN2), and protein phosphatase 1 (PP1) govern SG assembly. PP1 mediates SG dissolution and its role is conserved in mammalian cells, as judged by the enhancement of SG formation provoked by the PP1 inhibitor salubrinal. All these experiments were evaluated manually and by BUHO, and in all cases the differences were lower than 5% of the average value. The automated analysis of diverse cellular components by this user-friendly method allows massive image processing in short times as well as minimizes variations between different operators by providing a robust, flexible and reliable high-throughput alternative to the laborious and sometimes unfeasible visual scrutiny.

## Results

### Development of a MATLAB script for the identification of cells and SGs

**Figure 1:**
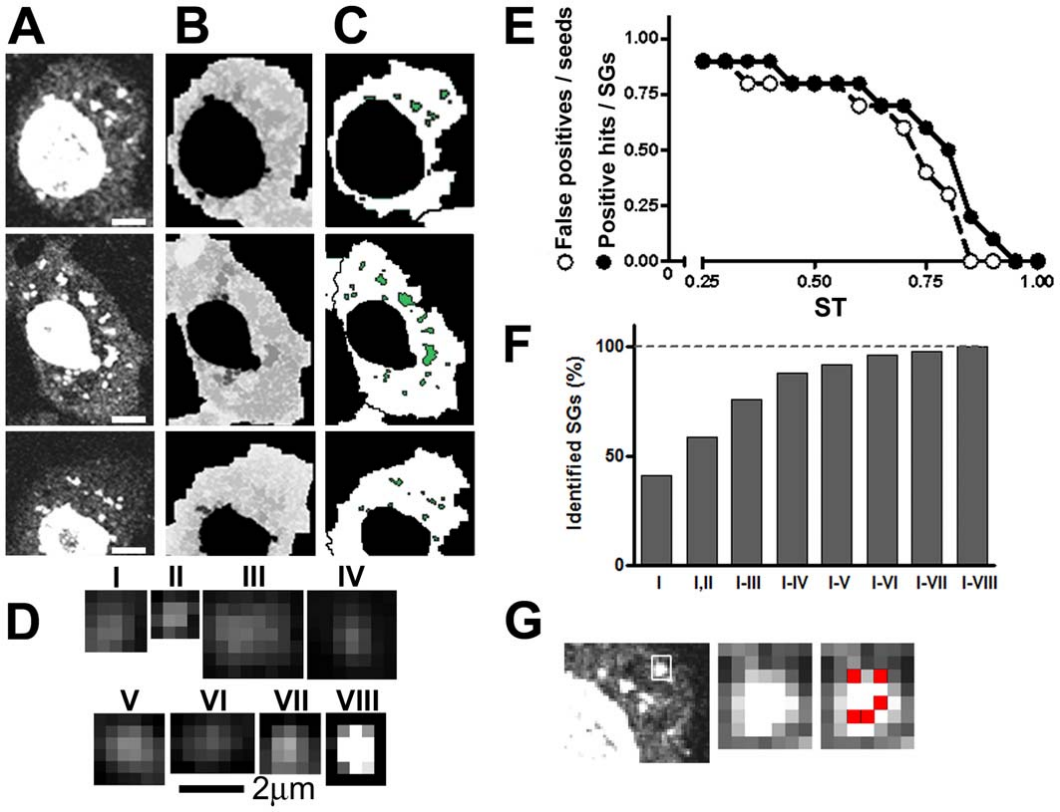
SGs in *Drosophila* S2R+ cells and script summary. Cells were grown, treated and stained in 384 MW plates as indicated in Materials and Methods. A, B and C, Three representative cells are shown. A, The oligodT-Cy3 allows the visualization of SGs in the cytoplasm and of accumulated polyadenylated RNA in the nucleus. B, Merged image created by MATLAB before application of the *Watershed* transform algorithm for the identification of cell border and nucleus. C. Objects were identified as SGs by correlation with the selected prototypes using the MATLAB *normxcorr2* function. D, Prototypes used to identify and analyze SGs. These were selected among 50 different examples to include differences in size, shape and intensity pattern. Size bar, 2 µm. E and F, A collection of 6 micrographs containing 125 cells with a total of 282 granules (see Table 1) were used to investigate the effect of varying the similarity threshold (ST) and of increasing numbers of prototypes. E, The proportion of false positives relative to the number of seeds and the proportion of positive hits relative to the manual-counted number of SGs was measured at different ST for the prototype IV, depicted in D. An ST of 0.85 allowed no false positives. SG recognition dropped from 80% to 20% at this ST value, and similar values were observed for the other prototypes (not shown). F, The number of positive hits that resulted by correlation with the indicated prototypes subsets was measured. All the SGs present in the micrographs were identified with the six prototypes depicted in D. Size bar, 5 µm. G, A representative SG from the cell in the first row showing the seeds (red) created by the script.

The accurate identification and characterization of cellular components using high-throughput microscopy, which retrieves a large amount of data, requires an automated methodology. To develop a MATLAB script for the computerized analysis of supramolecular aggregates we used SGs as a model system. These mRNA silencing *foci* are an ideal model since their size and number vary depending on the cellular physiology. *Drosophila* S2R+ cells were exposed or not to arsenite, a known inductor of oxidative stress that promotes SG formation [1], [33]. To visualize SGs and cell nuclei we co-stained the cells with oligodT-Cy3 and DAPI as indicated in Materials and Methods and 63× confocal images of 512×512 pixels were taken. As expected, in addition to SGs the FISH for polyadenylated RNA stained also the nucleus as well as faintly the cytoplasm (Figure 1A). Six images of stressed cells and three images of untreated cells were then manually analyzed. As previously reported [33], SGs formed in half of the cells (Table 1). These images were used as a training set to adjust a MATLAB script to identify cells and the SGs inside them. MATLAB imported the Cy3 and DAPI images as 512×512 matrices where each matrix element has values from 0 to 65535 corresponding to the intensity of each pixel in the16-bit images. Then, MATLAB operations were adjusted iteratively to minimize deviation from the manual analysis (Table 1), as described below.

**Table 1:** Summary of manual and script analysis of images used for BUHO adjustment.

Treatment	Cell number		SG number		Cells with SGs		SGs per cell	
	Manual	BUHO	Manual	BUHO	Manual	BUHO	Manual	BUHO
basal	18	18	0	0	0	0	0	0
basal	16	16	0	0	0	0	0	0
basal	21	21	0	0	0	0	0	0
stress 1	20	20	56	52	12	11	4.7	4.3
stress 2	20	20	40	40	11	11	3.6	3.6
stress 3	24	24	40	42	12	12	3.3	3.3
stress 4	18	18	12	12	5	5	2.4	2.4
stress 5	16	16	47	47	8	8	5.4	5.4
stress 6	27	27	87	89	17	17	5.1	5.2
Summary 1–6	**125**	**125**	**282**	**280**	**65**	**64**	**4.3**	**4.4**

Nine images from wells treated or not with arsenite (training set) were manually analyzed. Cells were considered positive when they contain two or more SGs [33]. Average number of SGs in the SG-positive cells is indicated. These values were used to tune the MATLAB script and values computed after BUHO optimization with this training set are indicated.

### Identification of cells by the watershed algorithm

**Figure 2:**
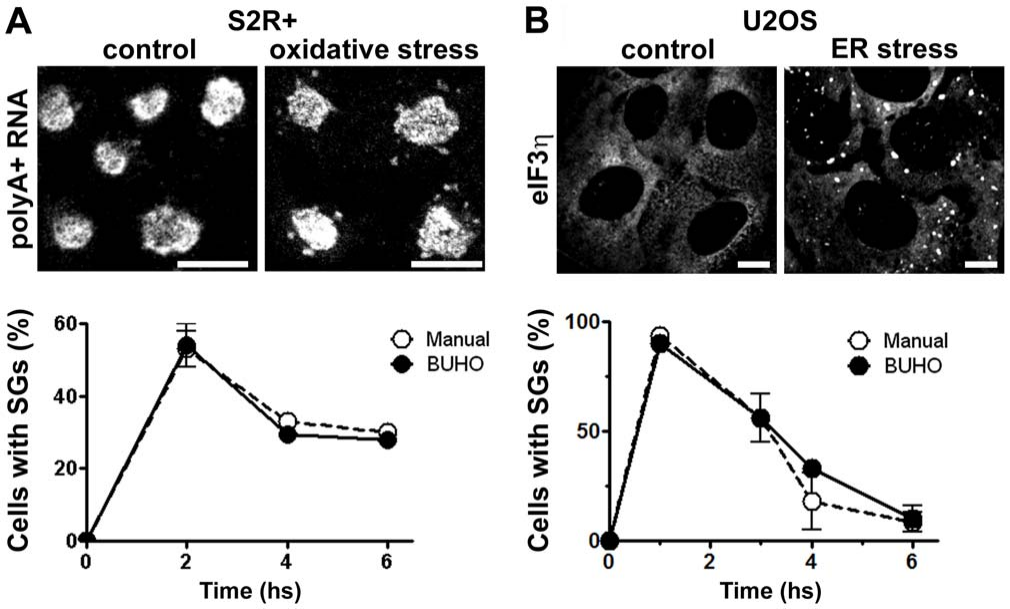
Time course of SG formation. A, *Drosophila* S2R+ cells were exposed to oxidative stress and SGs were visualized by FISH. B, ER-stress was induced in U2OS cells and SGs were visualized by immunofluorescence of eIF3η. Confocal images (63×, 1024×1024 pixels) were analyzed manually or with BUHO with a scaling down to 512×512. Ten micrographs from triplicate wells (A) and 5 micrographs from duplicates (B) were analyzed at each time point. Error bars indicate standard deviation. Size bar, 10 µm.

The first step was to identify the cells present in the micrographs. *Cell segmentation* was performed using the MATLAB *watershed transform algorithm* (*watershed* function). First, high-intensity noise was eliminated by setting a convenient threshold for each channel. Average DAPI nuclear intensity was 200 in the training set of images (available at https://sourceforge.net/projects/buho/) and less than 0.1% of DAPI-pixels reached values above 255. Cy3 intensities in SGs and cytoplasm were lower than 50 and less than 0.01% of the Cy3-pixels had values above 255. Thus, intensity was clipped to 255 in both the DAPI and the Cy3 channels. The contrast of the DAPI signal was adjusted with the *imadjust* function and the DAPI signal was used to create a nuclear mask image. All pixels with intensities lower than 60% of maximal values were converted to 0 and points above this threshold were converted to 1 with the *image to black and white* (*im2bw*) function. We combined the nuclear mask and the negative counterpart of the oligodT-Cy3 MATLAB matrices to generate a single image where all DAPI-positive pixels were converted to 0 and cells are brightly stained. Cells were clearly recognized in the image corresponding to this operation (Figure 1B). Next, to identify the cells by MATLAB, we calculated the *watershed lines* according to the *topographic distances method*. With this operation, the cell area corresponds to a *catchment basin* and the nuclei to its local minima or bottom of the *basin*. In summary, the nucleus acted as a marker for cell position and the inverted Cy3 intensity gradient in the cytoplasm allowed the identification of the cell border (Figure 1B, C). Previous to these operations, to compensate the faint overall cytoplasmic oligodT-Cy3 staining, values in the matrix corresponding to the Cy3 signal were multiplied by a factor of two, and additive noise was reduced using the *Wiener 2-D adaptive noise-removal filtering method* (*wiener2* function). These adjustments allowed for a better identification of the cell border, and were not required when the cytoplasm is strongly stained, as in the case of SG detection by immunofluorescence against eukaryotic initiation factor 3η (eIF3η) shown below (see Figure 2).

We observed that a reduced number of cells were irregular and smaller than average, with an area smaller than 30 µm^2^.These include partially attached cells, due to the semi-adherence of the S2R+ cell line, as well as a small number of unhealthy cells, and were not included in subsequent analysis.

### Identification of SGs by correlation with prototype granules

The next step was to identify the SGs present in the micrographs of the training set. This was done using a *normalized 2-D cross-correlation* between the matrix that correspond to the oligodT-Cy3 signal and several prototype SGs. Then, a set of 8 prototypes were selected among 50 different example SGs taken from the confocal images that represent the most frequent patterns of sizes, shapes and intensities: round, elliptical, with uniform intensity or with a brighter core (Figure 1D). Among other factors, we found that the inclusion of a margin of cytoplasm surrounding each prototype granule greatly improved their performance in identifying the SGs present in the original images. A preliminary analysis using Gaussian filters did not perform better than the prototypes selected from the micrographs.

For each combination of prototype and micrograph, a matrix was obtained containing correlation coefficients with values from −1 to 1, according to the similarity between the local pixel pattern and the prototype granule. Next, the points with correlation coefficients lower than a threshold termed similarity threshold (ST), which we empirically adjusted for each prototype (see below and Figure 1E) were eliminated using the *image to black and white* (*im2bw*) function, and a value of 0 was assigned to them. Points with correlation coefficients above the ST were assigned a value of 1. We called these points “seeds”. As expected, this operation generated a significant number of seeds inside the nucleus, as the abundant nuclear polyadenylated RNA is recognized by the Cy3-labelled probe (Figure 1A). Seeds inside the nucleus were eliminated, together with a small amount of seeds generated in extracellular regions.

We observed that a reduced number (7.5%) of SGs were weakly stained and were not recognized by the above operations. This was compensated by the application of the *imfilter* function with the *fspecial*: *unsharp* filter thus allowing faint or blurry SGs to be detected. These newly acquired SGs were added to the SGs identified in the unfiltered image, so for each micrograph, 16 correlations (8 prototypes, 2 Cy3-micrographs with or without filter) were assessed.

For each prototype, we investigated the effect of varying the ST (Figure 1E and data not shown). By analyzing the 6 representative images of the training set with a total of 282 SGs, we empirically determined that a ST of 0.89 for prototype I; 0.88 for prototype II; 0.8 for prototype III; 0.88 for prototype IV; 0.86 for prototype V; 0.86 for prototype VI; 0.92 for prototype VII; and 0.85 for prototype VIII; eliminated all false positives in each case. As expected, these strict ST values reduced the number of positive hits (Figure 1E and data not shown). This was compensated by the use of several prototypes. We found that with the above indicated ST, each prototype identified a fraction between 2% and 32% of the SGs present in the images. We found that with the 8 proposed prototypes (Figure 1D) the script recognized all the SGs present in the original micrographs of the training set (Figure 1F). However, as expected, a single SG was frequently recognized by more than one prototype (Figure 1G and Table 2). The whole process of nuclei, cell and SG identification is depicted in Figure S1.

**Table 2:** Redundant SG recognition by distinct prototypes.

Prototype	I	II	III	IV	V	VI	VII	VIII
**I**	41							
**II**	9	27						
**III**	3	2	21					
**IV**	1	2	5	20				
**V**	6	3	7	5	17			
**VI**	0	2	2	2	2	9		
**VII**	3	1	3	4	2	1	8	
**VIII**	2	0	1	2	2	1	3	6

The percentage of SGs recognized by each prototype, or simultaneously by pairs of prototypes in the 6 representative micrographs of the training set, with a total of 282 granules is indicated. ST was 0.89 for prototype I; 0.88 for prototype II; 0.8 for prototype III; 0.88 for prototype IV; 0.86 for prototype V; 0.86 for prototype VI; 0.92 for prototype VII; and 0.85 for prototype VIII. Redundancy allowed a single SG to be recognized by multiple prototypes.

SGs frequently display anisotropic staining and the selected prototypes are examples of anisotropic SGs. Then, we analyzed SG recognition by rotated versions of the prototypes depicted in Fig. 1D. Twenty four new prototypes were generated by three successive 90° clockwise rotations of the prototypes I to VIII, and their performance in SG identification was assessed at the same ST that the respective non-rotated prototypes. As expected, a variable fraction of SGs were identified by these 24 rotated prototypes (ranging from 2 to 32%). More important, we found that in all cases, the SGs identified by the rotated prototypes are redundantly identified by non-rotated prototypes (Figure S2). Thus, without losing sensitivity and to minimize the number of cross-correlations to be performed, rotated versions were not included in the proposed collection of prototypes.

The redundant recognition of a single SG leads to the assignment of more than one seed per SG. As a result, the SG will be counted more than once. This was corrected as follows: we applied a 3×3 dilation with the *imdilate* function to each seed to create a centered 3×3 square, which is the size of a small SG at this magnification and resolution (63×, 512×512 pixels), so that all the squares surrounding seeds in the same SG overlapped and became a single object. We confirmed that with this operation close SGs were identified as different objects. Finally, every object was reduced to a point with the function *bwmorph-shrink*, and the number of these points represented an accurate measurement of the number of SGs present in the original image. A combination of this matrix with that obtained by the *watershed transform* algorithm allowed calculating the number of SGs in each cell.

**Figure 3:**
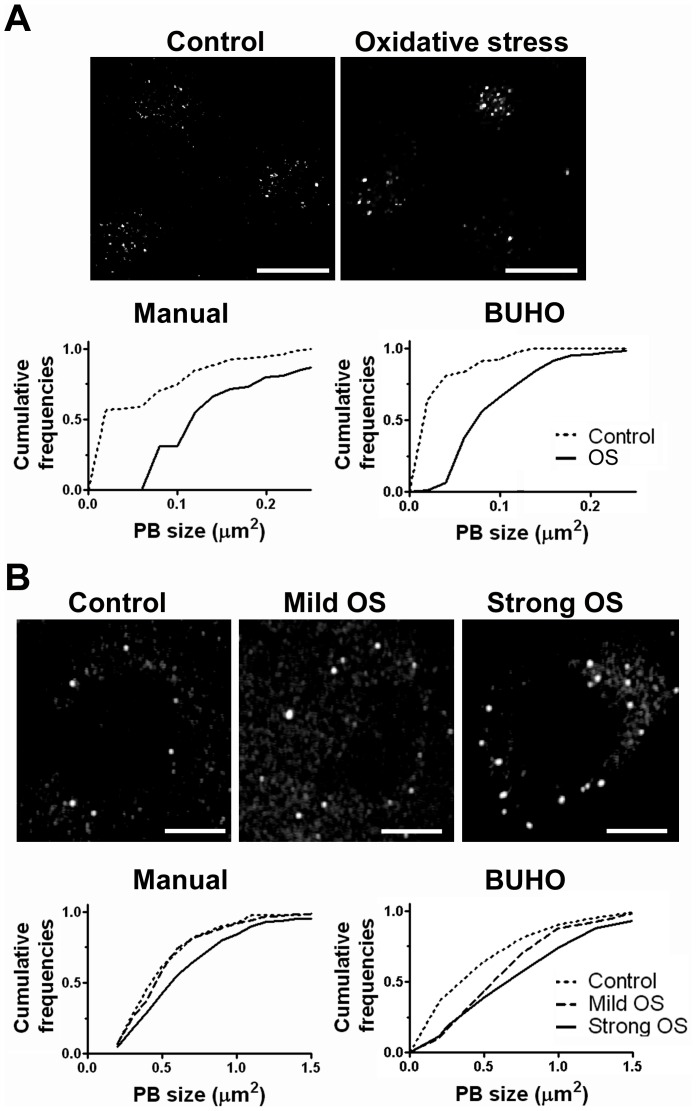
Analysis of PB induction by BUHO. Fly (A) or mammalian cells (B) were exposed to oxidative stress as indicated in Materials and Methods and PBs were immunostained. PB size was evaluated using BUHO in 55 resting and 68 stressed S2R+ cells, and in 15 resting and 23 stressed U2OS cells, and by manual counting in 10 stressed or 10 resting S2R+ representative cells and 9 stressed and 9 resting representative U2OS cells. 63×, 1024×1024 (A) or 40×, 2048×2048 (B) confocal images were used. Size bar, 10 µm.

As expected, the number of pixels in the above objects is a good estimation of the SG size in the original micrographs. However, we found that object size was better measured when it was above 6 pixels in width, thus higher resolution and/or higher magnification images are required for accurate measurements of granule size (see Figure 3 below). However, the object size in the micrograph is largely affected by the Point Spread Function of the objective and this may represent a serious limitation to size measurements by confocal microscopy. In summary, BUHO informs the cell number, the proportion of cells with SGs, the number of SGs per cell and their size with minimal deviation from manual-counted values (Table 1). In addition, the algorithm calculates the distances between equal or distinct objects stained simultaneously using the *bwdist* function.

### Example applications

To assess the suitability of BUHO for the analysis of additional cell structures, we investigated several examples of cytoplasmic *foci* stained with different strategies, including SGs and PBs in mammalian and insect cells, and synapses and Smaug 1-mRNA silencing *foci* (S-*foci*) in primary neurons. Finally, we used BUHO for the analysis of a pilot experiment aimed to identify new genes regulators of SG formation. In all cases, we compared the BUHO output with values obtained manually and found that differences were lower than 5% of the values.

### Example I: Time-course of SG formation analyzed by BUHO

SGs form transiently [1] and we analyzed the time-course of SG formation in *Drosophila* cells upon induction of oxidative stress. SGs in *Drosophila* S2R+ cells were visualized by FISH as described in Material and Methods (Figure 2A), the LSM images (63×, 1024×1024 pixels) were imported to MATLAB and scaled-down to 512 pixels using the *imresize* function. Then, cells and SGs were identified as described above using the following STs: 0.84, 0.88, 0.7, 0.8., 0.83, 0.8, 0.92, and 0.85 for prototypes I–VIII respectively. We compared the number of cells with SGs calculated by BUHO with the values obtained by manual analysis. Maximal formation was detected by both methods at 2 hs after the oxidative stress stimulus, with half of the cells showing SGs (Figure 2A). At all the time points analyzed BUHO and manual values diverge in less than 5% relative to the average values.

Next, we extended this study to the analysis of SGs induced in a different experimental system. We used mammalian U2OS cells exposed to thapsigargin, a known inductor of ER-stress, as indicated in Materials and Methods. To challenge our automated method with different data input, SGs were visualized by immunofluorescence against eIF3η, an accepted SG marker (Figure 2B). Images were taken at 63×, 1024×1024 pixels and scaled down to 512 pixels previous to analysis with the prototypes I–VIII as above. As before, BUHO allowed us to assess transient SG formation with high precision. Deviation from manually-counted values was less than 7% and both analysis showed a maximal response at 1 hour, when 93% of the cells had SGs. We concluded that BUHO perform successfully in the identification of SGs in cells stained by FISH against polyadenylated RNA or immunofluorescence against eIF3η. Moreover, the same 8 prototypes are suitable for SG identification in mammalian or *Drosophila* cells exposed to different insults.

### Example II: Analysis of PB induction upon stress

PB size increases upon cellular stress [1], and we used this as a model system to test the BUHO suitability to assess changes in *foci* size. *Drosophila* S2R+ or mammalian U2OS cells were exposed to oxidative or ER-stress as indicated in Materials and Methods, and PBs were visualized by immunostaining of the specific marker DCP1a (Figure 3A). *Drosophila* PBs were analyzed at 63×, 1024×1024 pixels, a resolution that we found suitable for size determination of objects with these dimensions (0.2–1 µm^2^/11–56 square pixels). Low intensity noise was removed with the *im2bw* function and prototypes I–VIII were compared at ST 0.8. Mammalian PBs were analyzed at 40×, 2048×2048 pixels. In this case, low intensity noise was removed and prototypes I–VIII were compared at STs 0.8, 0.88, 0.8, 0.8, 0.86, 0.86, 0.8, and 0.85 respectively. Adjustment of these parameters was performed by analyzing 123 representative S2R+ cells and 38 representative U2OS cells. Next, PB size was determined using BUHO and the manual method in parallel. As expected, both methods indicated that PBs were significantly larger after exposing the cells to oxidative stress (Figure 3A, B). *Drosophila* PBs enlarged by a factor of 3.5 and mammalian PB size by a factor 1.5 relative to their normal values. The difference of these values from manual parameters was less than 4% for basal PBs and 10% for induced PBs in *Drosophila* cells, and less than 7% for basal and 12% for induced PBs in mammalian cells. The total number of PBs in the micrographs was also measured, and we found that the BUHO-generated values (*Drosophila* basal, 350; *Drosophila* stress, 102; U2OS basal, 185; U2OS stress, 148) were comparable with the manual values (*Drosophila* basal, 309; *Drosophila* stress, 122; U2OS basal, 167; U2OS stress, 129). We concluded that BUHO successfully addresses changes in *foci* size and number. Moreover, as expected given that SGs and PBs are morphologically similar, we found that prototype SGs were useful to identify PBs.

### Example III: Dissolution of synaptic neuronal mRNA-silencing foci upon synaptic stimulation

**Figure 4:**
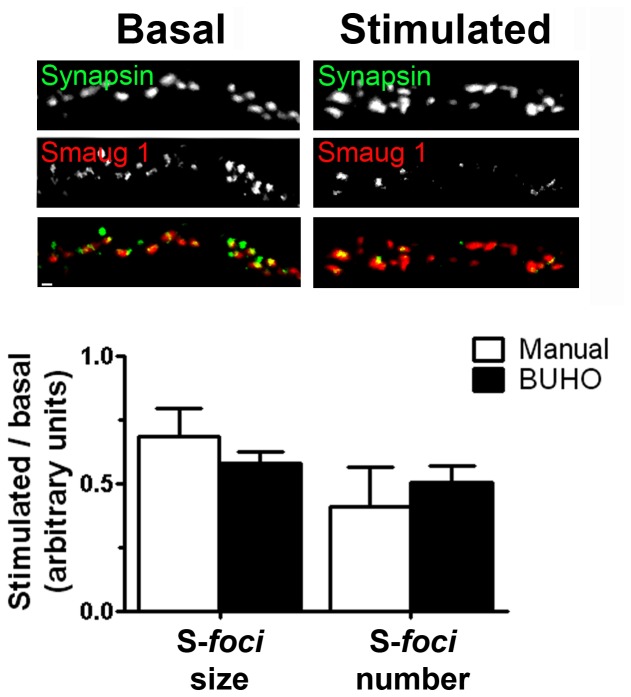
S-*foci* dissolution upon synaptic stimulation. Cultured rat neurons were stimulated with NMDA and S-*foci* and synapses were identified with specific antibodies as indicated in Materials and Methods. The S-*foci* size and the number of synapses containing S-*foci* in their surroundings at a distance lower than 0.5 µm were evaluated. S-*foci* smaller than 0.2 µm^2^ were not included. Six micrographs (63×, 1024×1024 pixels), containing 4180 synapses were analyzed using BUHO, and a subset of 200 representative synapses were analyzed manually. Values relative to basal conditions are plotted. Error bars indicate standard deviation. Size bar, 2 µm.

To test the performance of BUHO in analyzing distances between objects, we focused on the presence of synaptic mRNA silencing *foci* at the synapse surroundings. Briefly, neurons contain a plethora of mRNA-silencing *foci* collectively termed synaptic activity-regulated mRNA silencing *foci* (SyAS). Different synaptic stimuli enhance or reduce the presence of SyAS and there is an increasing interest in understanding SyAS dynamics [14], [15]. For instance, we recently found that a specific kind of synaptic *foci* named S-*foci* dissolve upon N-methyl-D-aspartate (NMDA) stimulation [14]. We studied the suitability of BUHO to assess this effect and compared it with the manual analysis. Primary neurons were exposed to NMDA as previously described [14] and S-*foci* and synapses were identified by immunostaining with specific antibodies as indicated in Materials and Methods. Using 63× micrographs, 1024×1024 pixels, we first evaluated S-*foci* size. The S-*foci* are morphologically similar to PBs ([14] Figure 4) and we used prototypes I–VIII to analyze them. As we did for PBs, low intensity noise was removed and prototypes were compared at ST 0.8. As previously reported, S-*foci* size was reduced to 71% of basal levels when obtained manually and to 61% when evaluated with the MATLAB script, which are comparable values [14]. We recently reported that in addition to this general reduction in size, synaptic stimulation by NMDA provokes the complete dissolution of the S-*foci* located at the synapse [14]. To evaluate this important local response we counted the number of synapses with S-*foci* in their surroundings. Synapses were stained with a specific antibody and identified by correlation with the prototypes I to VIII. Optimal ST was determined to be 0.7 in a preliminary analysis. We used the *bwdist* function to measure the distances between each synapse and the closer S-*foci*, and calculated the percentage of synapses with S-*foci* at a distance lower than 0.5 µm, which is physiologically relevant [14] (Figure 4). We found that the presence of S-*foci* at the synapse surroundings was reduced to 50% of basal values. When evaluated manually, they were affected in a similar proportion (41%, Figure 4). We concluded that BUHO is a reliable bioinformatics tool to identify synapses and to assess the vicinity between different cellular structures.

### Example IV: Identification of SG regulators by RNAi using BUHO

**Figure 5:**
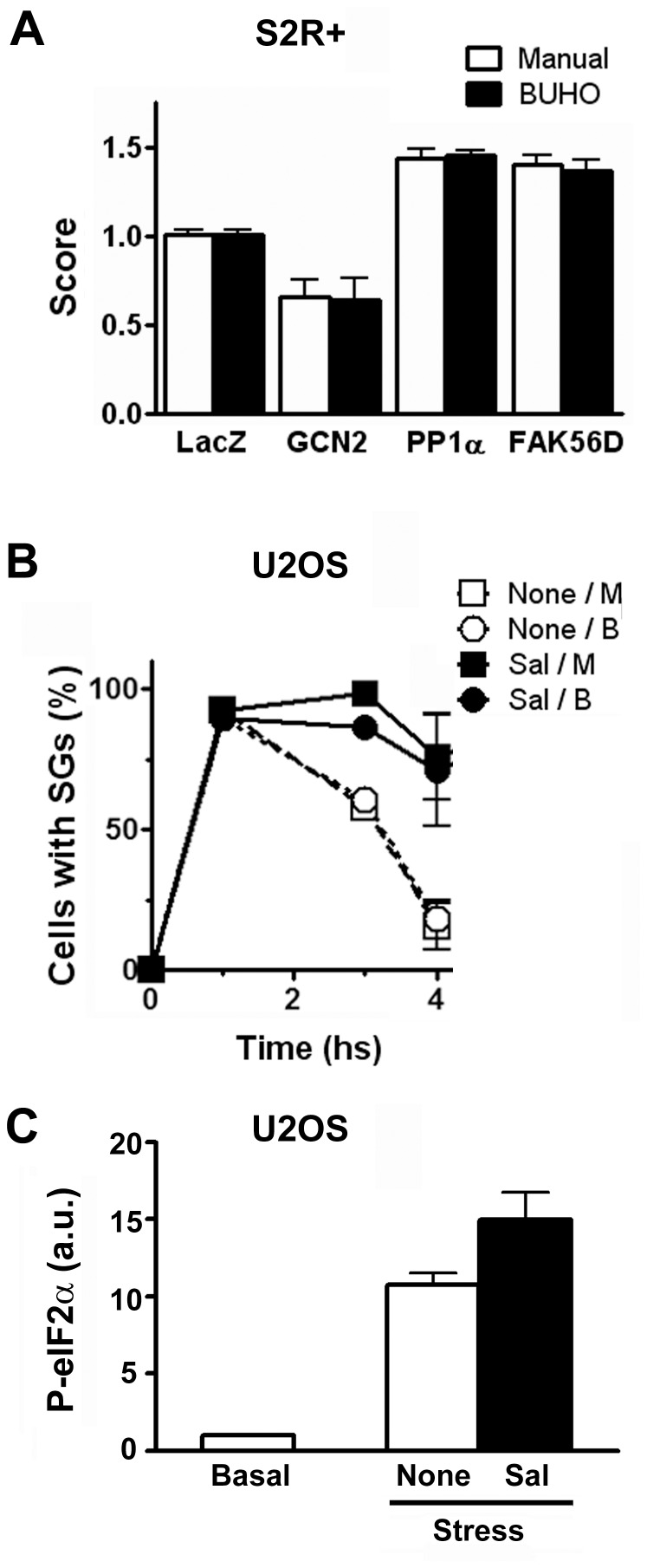
PP1α governs SG disassembly. A, *Drosophila* S2R+ cells were exposed to the indicated dsRNA and the effect on SG formation was evaluated. Triplicate wells of the indicated RNAi treatments were analyzed both manually and by BUHO. 204 control wells were analyzed by BUHO, and a subset of 6 wells (see Table 3) was analyzed manually. The score is the ratio of the percentage cells with SGs in each well relative to the average percentage of cells with SGs in the control wells in the same plate. GCN2 knockdown impaired SG formation, and the KD of FAK56D or PP1α-96A facilitated their assembly. Scores determined manually differ in less than 7% from those calculated by BUHO. B and C, Mammalian U2OS cells were exposed to ER-stress in the presence (Sal) or absence (none) of salubrinal, as indicated in Materials and Methods. B, The number of SG-positive cells at the indicated time points was evaluated manually (M) or by BUHO (B) in 63×, 1024×1024 images. Error bars, standard deviation. C, Phosphorylation of eIF2α relative to basal conditions was evaluated three hours after stress induction in the presence or absence of salubrinal, as indicated in Materials and Methods. Error bars, standard deviation.

Finally, to simulate a high-throughput RNAi screen, we used the BUHO script for the analysis of a pilot experiment aimed to identify SG regulators. We used *Drosophila* S2R+ cells in 384 MW plates with robotized manipulation for cell plating, stress induction, staining and imaging as indicated in Materials and Methods. We analyzed three candidate genes predicted to affect SG formation. Previous to the induction of oxidative stress, we treated the cells with dsRNA against FAK56D, GCN2, or PP1α-96A, or with a non-relevant dsRNA against LacZ. FAK56D is homologous to mammalian FAK, a kinase involved in SG disassembly [34]. GCN2 is an eIF2α kinase that mediates SG formation upon oxidative stress in *Drosophila* [35]. PP1α-96A is the main phosphatase of eIF2α and we speculated that this molecule affects SG stability. Triplicate plates including three well with a dsRNA for each gene, 20 wells with a dsRNA against LacZ and 68 wells with no dsRNA were analyzed. Seven images of each well were taken under the same conditions as those from the training set used for the script adjustment and analyzed manually and by BUHO using the prototypes I–VIII, as in Figure 2A. As expected, the values for the number of cells and SGs, and the proportion of cells with SGs were equivalent with the two methods (Table 3) when this test set of images was analyzed. We defined a score as the ratio of the percentage of cells with SGs upon dsRNA-treatment relative to the average percentage of cells with SGs in the controls without dsRNA present in the same plate. As expected, we found that the dsRNA against LacZ had no effect on SG formation (Figure 5A). As previously reported [35], GCN2 knockdown impaired SG formation. FAK56D knockdown had a stimulatory effect, as reported before for the mammalian counterpart [34]. In addition, we found that the knockdown of PP1α-96A enhanced SG formation in S2R+ cells. To investigate whether the role of this phosphatase is conserved in mammalian cells, we followed a pharmacological approach by using the PP1α inhibitor salubrinal. We exposed U2OS cells to a ER-stress insult in the presence or absence of salubrinal [36], which was added 1 hour after the stress-stimulus as indicated in Materials and Methods and investigated SG formation by immunostaining of eIF3η at different time points. SGs were identified by BUHO as in Figure 2B. We found that paralleling the effect of PP1α-96A knockdown in *Drosophila* cells the pharmacological inhibition of mammalian PP1α impaired SG disassembly in U2OS cells (Figure 5B). All these experiments were simultaneously assessed by manual analysis by an independent operator, and deviation from BUHO-obtained values was less than 5%. PP1 is the main eIF2α phosphatase and we evaluated phosphorylated eIF2α (PeIF2α) levels upon exposure to ER-stress in the presence or absence of salubrinal. As previously reported [1], [33], [37] we found that eIF2α phosphorylation increased 13 fold relative to basal values one hour after stimulation, when SG formation is maximal (Fig. 5B and data not shown). At this time, salubrinal was added to inhibit dephosphorylation and PeIF2α levels were monitored three hours after stress induction. Paralleling the prolonged occurrence of SGs in the presence of salubrinal, we found that PeIF2α levels were 50% higher in the presence of the PP1 inhibitor (Fig. 5C, p≤0.01). We speculate that dephosphorylation of eIF2α is key in regulating SG dissolution and this opens new lines of research. The success of BUHO in this pilot RNAi-based screen documented by automated microscopy underscores its potential in high-throughput imaging analysis.

**Table 3:** Summary of manual and BUHO analysis for the identification of SG regulators.

dsRNA	Cells with SGs (%)		SGs per cell	
	Manual	BUHO	Manual	BUHO
none	37	37	3.6	3.6
none	47	47	4.5	4.5
none	40	40	4.4	4.4
none	42	43	4.0	3.9
none	40	41	5.5	5.4
none	42	42	3.9	3.8
LacZ	42	42	4.2	4.2
LacZ	42	42	4.3	4.3
LacZ	41	43	4.7	4.8
GCN2	29	28	4.2	4.2
GCN2	37	38	4.4	4.4
GCN2	26	23	3.5	3.5
PP1α	59	58	5.0	4.9
PP1α	54	52	4.2	4.2
PP1α	53	57	4.3	4.3
FAK 56D	53	52	4.8	4.8
FAK 56D	57	54	5.1	5.1
FAK 56D	61	60	4.6	4.6

S2R+ cells were treated with the indicated dsRNA and exposed to arsenite. SG formation was analyzed in 7 images (test set) with an average of 300 cells for each treatment. Manual values and BUHO generated results are indicated.

## Discussion

In this work, we developed an original algorithm for the automated analysis of SGs and related cytoplasmic *foci*, and used it to identify PP1 as a novel regulator of SG formation. This MATLAB script, termed BUHO is easily commanded using Windows operating system and is based in the recognition by similarity to a collection of prototypes. The analysis of a pilot high-throughput experiment including 164304 images at 512×512 pixels was completed in 120 hours in a relatively low power computer (Perez-Pepe et al, unpublished). The script was optimized for the analysis of SGs in *Drosophila* cells, and is easy to adjust for the study of a wide variety of supramolecular aggregates in different cell types. With minor modifications, we have successfully applied it for processing images of different magnification and resolution of synapses, SGs, PBs and similar *foci* in mammalian and *Drosophila* cell lines as well as in primary cultured neurons. Remarkably, the deviation between BUHO and manual values was less than 5% of the average values. By using a different set of prototypes taken exclusively from confocal images or in combination with prototypes generated with Gaussian filters, we anticipate that BUHO will be also a valuable tool for the analysis of additional cellular structures and supramolecular aggregates that are emerging in the literature [17], [19], [20], [22], [23], [24].

To further validate the multiple uses of this bioinformatics method, we utilized BUHO in the analysis of a pilot experiment aimed to investigate the effect of candidate genes on SG dynamics. As expected, we found that this script is a reliable tool to assess changes in SG formation. As reported before for the mammalian FAK56D homologue, we found that the knockdown of this kinase in *Drosophila* cells enhances SG formation, whereas down-regulation of GCN2 impairs their assembly [34]. In addition, we found that the catalytic subunit of PP1 mediates SG dissolution. The role of this main phosphatase appeared conserved in flies and mammals, and furthermore, we found that PP1 affected the disassembly of SGs induced by different stress stimuli. Phosphorylation of eIF2α provokes translation inhibition and concomitant SG formation, and dephosphorylation of eIF2α correlates with translation recovery after acute stress [1]. Accordingly, our results indicate that PP1 mediates eIF2α dephosphorylation, and we speculate that this plays an important role in SG stabilization upon PP1 KD or inhibition by salubrinal. The relevance of additional PP1 targets involved in SG dissolution is unknown. Salubrinal is a relatively new drug and its potential as therapeutic agent is expanding [36]. The contribution of SG regulation to the beneficial effect of this inhibitor remains to be investigated.

We anticipate that the high-throughput screening of natural or synthetic compounds will benefit from the automated analysis of SG formation described here. SGs always form when cells are exposed to stress insults and their formation will be indicative of cell toxicity, which is an important effect to assess when analyzing novel chemicals. The use of BUHO for the automated analysis of other mRNA silencing *foci*, protein aggregates and cytoplasmic structures in addition to SGs, PBs, synapses and S-*foci* will require only minor modifications. The spontaneous formation of microscopically visible aggregates that concentrate molecules involved in distinct pathways is common in many different cell types. A fraction of a yeast library including thousands of genes with C-terminal GFP-fusions was visually screened to reveal that numerous proteins form filaments and *foci* [20], [22]. The usefulness of these libraries will be greatly potentiated by high-throughput image analysis like the one described here. Purinosomes, glutamine synthetase *foci* and other supramolecular factories optimize the biosynthetic pathway by channeling substrates, minimizing diffusion to the cytosol and protecting labile intermediates [19], [20]. Conversely, a number of enzyme aggregates appears to serve as depots [22]. In addition to enzyme aggregates, UV-RNA granules, Ire-1 *foci*, splicing speckles, nuclear stress bodies, Cajal bodies, eIF2B bodies, and other cytoplasmic or nuclear structures assemble and disassemble dynamically depending on cellular needs. The script here described will result a suitable tool for the high-throughput analysis of these different supramolecular aggregates in diverse experimental settings. Among other outputs, BUHO addresses the number, size and distance between objects, and categorize them by similarity in shape, size and intensity to a variable number of prototypes isolated from original images. By adjusting the correct parameters BUHO can be adapted to the automated analysis of many different cellular components. This user-friendly script allows the analysis of high-throughput imaging data in short times and eliminates variations associated to tedious manual scrutiny that frequently involves more than one operator. This novel bioinformatics tool is a step forward in the expanding field of cellular supramolecular aggregates.

## Materials and Methods

### Stress induction, drugs and dsRNA treatment of Drosophila and mammalian cells

*Drosophila* S2R+ cells were grown in 384 MW plates (Figure 1 and 5, Table 1, 2, and 3) or in 10 mm glass coverslips (Figure 2 and 3), as previously described [33]. Oxidative stress was induced by exposure to 500 µM arsenite during 2 hs unless otherwise indicated. When required, cells in 384MW plates were treated with 0.25 µg dsRNA during 3 days, as is routine at the *Drosophila* RNAi Screening Center (DRSC), Harvard Medical School. We used the following dsRNAs, designed by the DRSC: DRSC16678 against GCN2, DRSC16795 against PP1α-96A and DRSC07426 against FAK56D. All manipulations, including plating, treatment and staining in 384 MW plates were performed in a robotized system at the DRSC.

U2OS cells from the American Type Culture Collection (ATCC) were grown on 10 mm glass coverslips in DMEM (Sigma) as before [33], [37]. When indicated, 100 nM thapsigargin (Sigma) was added to conditioned media, and 25 µM salubrinal (Calbiochem EMD Bioscience, San Diego, CA) in DMSO or DMSO alone as control were added one hour afterwards. Mild oxidative stress was triggered with 250 µM arsenite plus 100 µg/ml puromycin and strong oxidative stress with 500 µM arsenite plus 250 µg/ml puromycin during one hour, as previously described [38]. Primary hippocampal neurons were prepared and treated with 30 µM NMDA (Sigma) during 5 minutes as previously described [14].

### Fluorescent in situ hybridization (FISH) and immunofluorescence

SGs in *Drosophila* S2R+ cells were visualized by FISH for polyadenylated RNA using oligodT-Cy3 (Sigma), as previously indicated [33] and with automated processing for the 384 MW plates. Immunofluorescence of *Drosophila* and mammalian cells was performed after fixation, permeabilization and blocking as previously done [14], [33], [39]. Primary antibodies were used as follows: goat anti-eIF3η (Santa Cruz); 1∶200; mouse anti-Dcp1a (Abnova), 1∶500; rabbit anti-PeIF2α (Cell Signaling), 1∶100; anti-mammalian Smaug1, 1∶500 [40]; IgG1 anti-synapsin (Synaptic Systems), 1∶250. The secondary antibodies used were: anti-goat Cy5 (Jackson Immuno Research Laboratories), 1∶200 and anti-mouse alexa 488 (Molecular Probes), 1∶500, anti-rabbit Cy3 (Jackson Immuno Research Laboratories), 1∶200. Nuclei were visualized by DAPI staining. Cells grown in glass coverslips were mounted in Mowiol 4–88 (Calbiochem, EMD Bioscience, San Diego, CA).

### Confocal imaging and analysis

Cells in 384 MW plates were analyzed in an OPERA Autoscope at the DRSC and 63× micrographs were captured at 512×512 pixels. Cells in glass coverslips were analyzed after mounting. Images were acquired in a LSM510 Meta confocal microscope (Carl Zeiss, Oberkochen, Germany) using Zeiss LSM software at 25°C and EC ‘Plan-Neofluor’ 40×/1.30 Oil or a Plan-Apochromat 63×/1.4 Oil. Equipment adjustment was assessed using 1 µm FocalCheck fluorescent microspheres (Molecular Probes). Magnification and resolution were as follows: Figures 2A, 2B, 3A, 4 and 5B: 63×, 1024×1024 pixels; Figure 3B: 40×, 2048×2048 pixels; Figure 1A and 5A: 63×, 512×512 pixels.

Manual and BUHO analysis were carried out independently by two different operators. Cells and particles were manually analyzed by Image J (NIH). Signal intensity was analyzed by Image J. LSM or TIFF format images were used for the analysis with MATLAB (MATrix LABoratory, The Mathworks Inc.), version R2008a. The *bwdist; bwmorph; imadjust; imcrop; imdilate; imfill; imfilter; imread; regionprops; watershed and wiener2* functions from the MATLAB Image Processing Toolbox were used. Monochromatic images correspond to a 512×512, 1024×1024 or 2048×2048 MATLAB matrix, where the intensity of each pixel is a value ranging from 0 to 255 for 8-bit images (Figures 2, 3, 4 and 5B), or from 0 to 65535 for 16-bit images (Figures 1 and 5A). When indicated, resolution was scaled down to 512 pixels. The MATLAB script developed here, termed BUHO and the confocal images are available at https://sourceforge.net/projects/buho/. The proposed collection of prototypes is available at PROTOTYPE.rar at the same URL.

## Supporting Information

Figure S1 BUHO and manual analysis of cells and SGs. A representative region from the training set of images (see Table 1) is depicted through the whole process of nucleus, cell and SG identification. A, the DAPI staining is converted to a nuclear mask using the *im2bw* MATLAB function (*image to black and white*). The oligodT-Cy3 staining was negatively converted with the *imcomplement* function (*image complement*). The two matrices were combined to generate an image with black nuclei and bright cytoplasm. Then, the *watershed transform algorithm* (*watershed* function) was used to delimitate the cell borders. B, The oligodT-Cy3 image was used to detect SGs similar to Prototype I. The pixel pattern of each pixel of the Cy3 image is compared with the pixel pattern of Prototype 1 by a *normalized 2-D cross-correlation (normxcorr2)*, which generate an image where each pixel has a value from −1 to 1, with 1 indicating the higher similarity. Then, a similarity threshold empirically adjusted to avoid false positives (see text) is applied and pixels with values higher than ST = 0.89 are assigned a value of 1. These operations generate seeds in each hit. Finally, the *imdilate* function convert the seeds into larger objects, the identified SGs. C, Identification of SGs by prototypes I to VIII. A *normalized 2-D cross-correlation* was performed for each prototype. The prototype I identifies 6 different SGs (yellow arrows). The prototype II identifies two additional SGs (blue arrows). The prototype III identifies seven SGs (red arrows), one of them previously detected by prototype II (left upper corner). The prototype IV identifies five SGs (orange arrows), only one of them previously undetected (second cell from right). The prototype V identifies three SGs (green arrows) previously detected by prototypes I, II and III, respectively. The prototype VI identifies two new SGs and two SGs already detected by prototype III (pink arrows). The prototypes VII and VIII don't match any SG in this image, according to their low identification rate (Figure 1F and Table 2). See text and BUHO_Process at https://sourceforge.net/projects/buho/ for more detail.
(TIF)

Figure S2 Redundant identification by rotated prototypes. Prototypes I–VIII depicted in Figure 1F were rotated 90, 180 or 270 degrees clockwise using the *imrotate* function and used as probes to detect SGs in the training set of images. The subsets of SGs recognized by each rotated prototype was then tested against non-rotated prototypes I to VIII in an additive manner (against prototype I only; against prototype I and II, et cetera). In all cases 100% of the SGs identified by rotated prototypes were redundantly recognized by the collection of non-rotated prototypes.
(TIF)
