# Supplementary figures and images for: BUHO: A MATLAB Script for the Study of Stress Granules and Processing Bodies by High-Throughput Image Analysis
Source: PLoS One. 2012 Dec 20;7(12):e51495. doi: 10.1371/journal.pone.0051495 (PMC3527446; doi:10.1371/journal.pone.0051495)

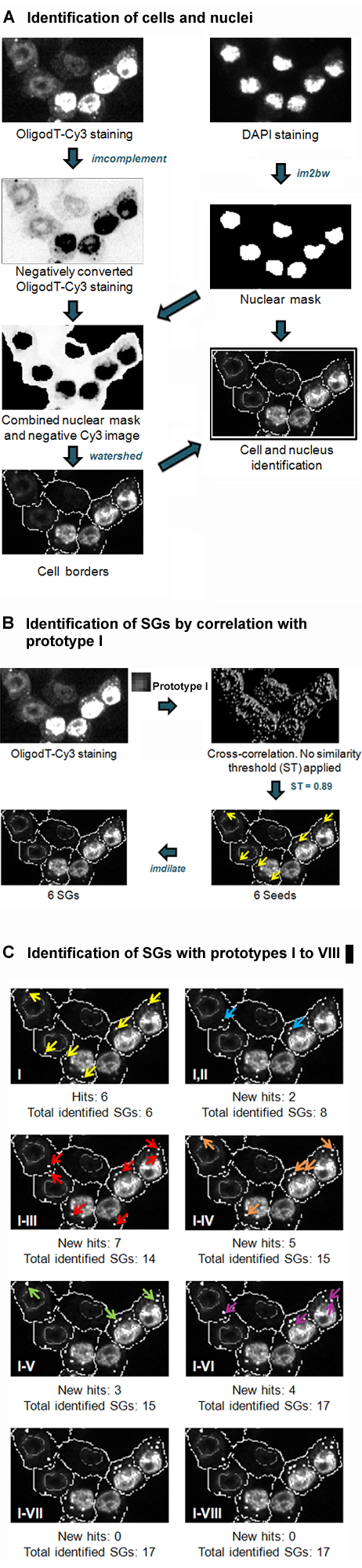

Supplement: Figure S1 — BUHO and manual analysis of cells and SGs. A representative region from the training set of images (see Table 1) is depicted through the whole process of nucleus, cell and SG identification. A, the DAPI staining is converted to a nuclear mask using the im2bw MATLAB function (image to black and white). The oligodT-Cy3 staining was negatively converted with the imcomplement function (image complement). The two matrices were combined to generate an image with black nuclei and bright cytoplasm. Then, the watershed transform algorithm (watershed function) was used to delimitate the cell borders. B, The oligodT-Cy3 image was used to detect SGs similar to Prototype I. The pixel pattern of each pixel of the Cy3 image is compared with the pixel pattern of Prototype 1 by a normalized 2-D cross-correlation (normxcorr2), which generate an image where each pixel has a value from −1 to 1, with 1 indicating the higher similarity. Then, a similarity threshold empirically adjusted to avoid false positives (see text) is applied and pixels with values higher than ST = 0.89 are assigned a value of 1. These operations generate seeds in each hit. Finally, the imdilate function convert the seeds into larger objects, the identified SGs. C, Identification of SGs by prototypes I to VIII. A normalized 2-D cross-correlation was performed for each prototype. The prototype I identifies 6 different SGs (yellow arrows). The prototype II identifies two additional SGs (blue arrows). The prototype III identifies seven SGs (red arrows), one of them previously detected by prototype II (left upper corner). The prototype IV identifies five SGs (orange arrows), only one of them previously undetected (second cell from right). The prototype V identifies three SGs (green arrows) previously detected by prototypes I, II and III, respectively. The prototype VI identifies two new SGs and two SGs already detected by prototype III (pink arrows). The prototypes VII and VIII don't match any SG in this image, accordi [file pone.0051495.s001.tif]
